# The effect of cryoprecipitate on prevention of intra cerebral hemorrhage and brain contusion expansion in traumatic patients

**DOI:** 10.5249/jivr.v14i3.1763

**Published:** 2022-07

**Authors:** Hamidreza Saeidiborojeni, Alireza Tahmoures, Mehdi Naderi, Mohammad Reza Akrami

**Affiliations:** ^*a*^ Department of Neurosurgery, School of Medicine, Kermanshah University of Medical Sciences, Kermanshah, Iran.; ^*b*^ Clinical Research Development Center, Taleghani and Imam Ali Hospital, Kermanshah University of Medical Sciences, Kermanshah, Iran.

**Keywords:** Contusion, Traumatic brain injury, Cryoprecipitate

## Abstract

**Background:**

Traumatic brain injury (TBI) is a general and socioeconomic complication and is one of the im-portant causes of mortality and disability among young people in the world. Falling and vio-lence and sports injuries are the other cause. It causes for about ten million new patients, accounting for 9% of all deaths. This interventional study aims to investigate the effects of early administration of cryoprecipitate to prevent expansion of intracranial hemorrhage.

**Methods:**

This randomized clinical trial recruited 54 non-pregnant patients. 27 patients in the control group and 27patients in the interventional group. For all patients, common and accepted procedures in scientific centers, including anticonvulsant drugs, normal saline and the other routine management was done and only for patients in the intervention group, 4 units of cryoprecipitate were added to their routine treatments; computed tomography scan (CT) scan was performed 48 hours later in both groups and finally the contusion size was compared in both groups.

**Results:**

It was observed in the intervention group that by adding 4 units of cryoprecipitate to their treatments; they had no increased size of the brain parenchymal contusion according to the criteria defined in the study compared to the control group (OR: 0.08, 95% CI: 0.0102_0.6303)

**Conclusions:**

According to a clinical trial, it seems that cryoprecipitate can prevent of cerebral parenchymal hemorrhage expansion in traumatic patients.

## Introduction

Traumatic brain injury (TBI) is a critical public health and socioeconomic complication in the world. It is one of the most important reasons of death, particularly among young adults.^1^ However high-quality prevalence information is rare, it is assessed that in the USA, around 5.3 million persons are living with a TBI-related disability, and in the European Union, approximately 7.7 million persons who have experienced a TBI have disabilities.^2,3^

TBI usually leads to neurological deficits (such as attention deficit disorder, failure to form visuospatial associations, or poor executive function) and mental health issues.^4^ It can cause cerebral edema, contusion or intracerebral hemorrhage parenchymal and non-parenchymal which leads to cerebral hernia by increasing intracerebral pressure. Blood vessel damage that occurs due to the spread of bleeding in the perivascular surface activates a cascade of events that causes hemorrhage and destroys the blood-brain barrier and results in secondary complications. Immediately after the initial injury, brain edema, the formation of free radical, and inflammatory mediators releasing leads to secondary brain damage, that is the main cause of death and disability.^5,6^ If the patient survives the initial injury, the small contusion will be completely absorbed within 2 to 3 weeks. Secondary damage occurs due to a pronounced inflammatory response to the presence of intracerebral hematoma. Activation of the systemic inflammatory response causes exacerbation of vasogenic edema.^7^

Blood coming out of a blood vessel is neurotoxic, and hematoma products, including hemoglobin and Fe++, may be involved in the development of cerebral edema, cell death and long-term damage of tissue. Hematomas caused by increased intracerebral pressure cause adverse effects. Fibrinogen is the first coagulation factor that is decline in the acute phase, due to becoming fibrin, leading to the development of coagulopathy.^8^

Three important sources of fibrinogen supply include FFP, cryoprecipitate and fibrinogen concentrate.^9^ Cryoprecipitate containing fibrinogen, Factor 8, factor 13 and VWF, which can rapidly increase fibrinogen levels.^10^

Fibrinogen is also involved in platelet aggregation, and factor 13 in cryoprecipitate binds fibrin monomers from fibrin degradation.^11^ Because cryoprecipitate has a factor of 8, it can also activate internal clotting pathways, so it has the ability to prevent hematoma enlargement.^10^

There is a possibility that in traumatic brain injury, intra cerebral hemorrhage and brain contusion may increase by 25 to 51 percent in the first 24 hours after the accident, resulting in a higher surgical rate, increased disability, or even death and mortality. If we can prevent the spread of hemorrhage after trauma, further damage to brain tissue can be prevented. Therefore, prevent the spread of hemorrhage after trauma can reduce the mortality, disability, and surgical procedures. In this study we decided to examine the effect of cryoprecipitate on prevention of intra cerebral hemorrhage and brain contusion expansion in traumatic patients.

## Methods 

The present study is a double-blind clinical trial with parallel groups that was conducted in Kermanshah Taleghani Hospital, at population after traumatic hemorrhage in the parenchymal tissue. The population consisted of patients with brain injury who were randomly divided into two groups. Before the start of the study, the participants in the study were given informed consent by the legal guardians of each patient and upon complete information, they agreed to participate in the study for the patient. Fifty-four patients participated in this study. 27 patients in the control group and 27 patients were in the intervention group. In the intervention group, in addition to standard measures, including administration of antiepileptic drugs, normal saline, and close control of the level of consciousness, four units of cryoprecipitate were prescribed in the first period after confirming the contusion or hematoma in the CT scan. CT scans were performed for both groups based on routine CT scans of the brain. After the initial CT scan, sonography was performed and the size of the hematoma was assessed separately. It should be noted that the procedure should be interrupted in case of unjustifiable complications after taking cryoprecipitate, which could harm the treatment or the patient's health.

## Results

This study was performed on 54 patients who were selected through random selection. Patients were randomly divided into two groups including intervention (n = 27) and control group (n = 27) (Table 1).

**Table 1:** Frequency distribution of variables

Variable	Control Mean ± SD	Treatment Mean ± SD
Age (years)	42.96±17.56	42.81±16.21
GCS	13.18±1.88	13.66±1.38
BMI	23.74±2.47	23.68±2.51
Primary bleeding size (mm)	7.46±6.17	20.55±19.88
Bleeding size after 48 hours (mm)	15.97±17.95	26.32±36.36
Blood sugar (BS) (mg/dl)	138.74±29.20	132.70±30.67
Blood platelets (*1000)	235.96±(44.04	227.33±58.38
INR	1.02± 0.103	1.04± 0.105
PT (sec)	12.85±0.60	13.14±0.81
PTT (sec)	33.92±4.06	33.70±3.71

In terms of gender fit in the control group were 17 males (62.96) and 10 females (37.04) and in the treatment group included 18 males (66.67) and 9 females (33.33) (Table 2 ).

**Table 2:** Gender frequency.

Variable Gender	Control N (%)	Treatment N (%)
Male	18 (66.67%)	17 (66.67 %)
Female	9 (33.33%)	10 (37.04%)
Sum	27 (100%)	27 (100%)

The GCS of patients in the control and treatment groups is shown in Table 3. In GCS classification, based on severity, 22 patients in the control group were in the Mild range and 4 patients were in the Moderate range and one patient was in the Sever range. In the intervention group 23 patients were in the Mild range and 4 patients were in the Moderate range, no cases were observed in the intervention group in the Sever range. The results of body mass index in the control and treatment groups are summarized in Table 3.

**Table 3:** Frequency of patients based on the severity of GCS and BMI.

Variable		Control N (%)	Treatment N (%)
**GCS **	Mild	22 (81.48%)	23 (85.19%)
	Moderate	4 (14.81%)	4 (14.81%)
	Sever	1 (3.70%)	-
	BMI	N (%)	N (%)
	Normal	20 (74.07%)	21 (77.78%)
**BMI**	Over weight	6 (22.22%)	6 (22.22%)
	Obese	1 (3.70%)	-
	Sum	27 (100%)	27 (100%)

In the study of blood sugar, patients in each of the control and treatment groups had 3 patients with diabetes mellitus (11.11). The results for blood sugar are presented in Table 4.

**Table 4:** Frequency of diabetes and coagulation disorder.

Variable		Control N (%)	Treatment N (%)
**Diabetes **	Positive	3 (11.11%)	3 (11.11%)
	Negative	25 (88.89%)	24 (88.89%)
**Coagulation disorder**	Positive	1 (3.70%)	2 (7.41%)
	Negative	26 (96.30%)	25 (92.59%)

In the control group, one patient (3.70%) and in the intervention group, 2 patients (7.41%) showed coagulation disorders based on the results of the requested tests (PT and PTT), and no patient mentioned the use of anticoagulants in either of the two control and intervention groups. Patients' platelets were calculated between 167000-315000 in the control group and between 154000- 358000 in the intervention group (Table 4 ).

The distribution of bleeding based on the location of the contusion in the control and treatment groups is summarized in (Table 5). Increase in bleeding size according to the criteria defined in the study after the first 48 hours of admission was observed in 11 patients in the control group (40.74%) and in the intervention group in 4 patients (14.81%) (Table 6).

**Table 5:** Frequency of bleeding sites in brain of patients.

Variable Place of bleeding	Control N (%)	Treatment N (%)
Temporal	10 (37.04%)	13 (48.15%)
Frontal	12 (44.44%)	10 (37.04%)
Parietal	4 (14.81%)	3 (11.11%)
Occipital	1 (3.70%)	1 (3.7%)
Sum	27 (100%)	27 (100%)

**Table 6:** Frequency of number of people with increased bleeding.

Variable Diabetes	Control N (%)	Treatment N (%)
Positive	11 (40.74%)	4 (14.81%)
Negative	16 (59.26%)	23 (85.19%)
Sum	27 (100%)	27 (100%)

**Table 7:** Evaluation of logistic regression in patients in the treatment group.

Variable	Odds coefficient	Standard error	P value
Increase the size	0.080	0.084	0.016
Age	1.04	0.027	0.08
Gender	1.81	1.57	0.49
GCS	0.85	0.224	0.55
BMI	0.74	0.166	0.18
Diabetes	4.51	6.0	0.25
Coagulation disorder	2.45	3.99	0.58
Bleeding size after 48 hours	1.00	0.002	0.03

## Discussion

Bleeding in the parenchymal tissue of the brain contusion and intra cerebral hemorrhage (ICH) is a complication of traumatic brain injury (TBI) that can occur following an accident, fall, conflict, or exercise. In TBI, the risk of brain damage is reported to be up to 42%, of which 3% are contusion and 10% are ICH. Most TBI-induced contusions are located in the sub-frontal and temporal regions. Following contusionesis in at least 25% of cases, during the phenomenon of Bloosming, we have an increase in the size of the brain parenchymal tissue hematoma, which itself can increase mortality and morbidity. Recent studies indicate the development of acute coagulopathy in 25% to 30% of patients with acute trauma, including TBI. Therefore, early correction of coagulation disorder is one of the important goals of treatment in TBI, which is related to survival and should begin immediately from the moment of admission to the hospital. The cause of coagulopathy in these patients is often multifactorial and includes various cases such as: loss of coagulation factors during site bleeding or dilution coagulopathies following IV Therapy or PRBC administration and so on. Other factors reducing coagulation factors include fibrinolysis activation, alternative pathway activation, acidemia, hypothermia, and shock. Dilutional coagulopathy is now recognized as a key element of trauma-related coagulopathy. The rate of coagulopathy from 1% in mild cases to 6% in cases of sever TBI has been reported in various studies and the increase in coagulopathy has been associated with an increase in the severity of TBI. The presence of coagulopathy in TBI is associated with the development of a hemorrhagic lesion, which is not limited to the expansion of the existing contagion but can cause new lesions in different locations in the parenchyma. This increase in hematoma size is more common in the elderly, and existing coagulopathy is associated with increased mortality and more adverse outcomes. It should be noted that screening tests used to measure coagulopathy (PT and PTT) in all cases where the homeostasis is abnormal can be reported normally, and for them to be reported abnormal, it takes time and a large amount of coagulation factors to be lost. Due to the effective role of coagulopathy in causing cantogenesis and increasing the size of cerebral hemorrhages, various studies have been performed to reduce the complications of TBI by preventing the development and spread of coagulopathy. The most common options used to treat coagulation disorders are blood components and factors, including FFB, platelets, cryoprecipitate, natural or synthetic blood concentrates (such as recombinant factor VIIa). In this study, the efficacy of cryoprecipitate on patients with cerebral contusion was investigated. Thus, 54 TBI patients who were diagnosed with brain contusion were randomly assigned to two groups of 27 patients. For the control group, accepted routine procedures were performed according to the available guidelines, and for the other group, in addition to these measures, 4 units cryoprecipitate blood transfusion was on the agenda at the first opportunity after diagnosis. The size of the first hematoma diagnosed was measured at the time of admission and patients in both groups were monitored for 48 hours. After 48 hours, CT was requested again for both groups. Effective on increasing hematoma size including: age, sex, platelet count, diabetes and BMI were controlled by random selection of these interfering factors. The results showed that in the intervention group, by administering 4 units of cryoprecipitate, the increase in hematoma size after 48 hours of injury was 92% less than the control group. It should be noted that no complications were observed in the administration of cryoprecipitate in the intervention group. Traumatic brain injury is accompanied by acute and chronic changes in neural functions^11^ and is known as a complex and multidimensional nerve injury.^12^

Both erythropoietin, a glycoprotein hormone with pleiotropic cytokine-like activities, and its receptor exist in the central nervous system.^13^ Erythropoietin and its receptor could be also found in the peripheral nervous system.^14^ Although erythropoietin and its receptors are present in the normal adult brain, their level will increment in neurons, glial, neuronal progenitor cells, and endothelial cells in response to injury.^15^ Elevated erythropoietin rates are also found in Schwann cells following peripheral nerve damage.^14^ Evidence suggests that peripherally administered erythropoietin can pass across the blood-brain barrier via especial transport mechanisms which might be modulated during cerebral hypoxia.^16^

Investigations have shown that erythropoietin improves neurological results after traumatic brain injury, thus it could be a potential candidate to improve secondary brain damage in traumatic brain injury.^17^ Previous studies clarified that, in patients with acute TBI, administration of erythropoiesis-stimulating factor can significantly improve the survival rate with no rise in morbidity.^18^ Previous studies have not performed any clinical trials on the effect of cryoprecipitate on brain contagions, and only a 2015 study by Alexander Olason et al. In Australia found that in 156 patients with major trauma, those transfused blood products with cryoprecipitate had lower isolated TBI and less GCS than those who received blood products without cryoprecipitate and experiments showed that they had hypofibrinogen.^19^ An in vitro study by Cushing et al. showed for the first time in the laboratory that cryoprecipitation has the ability to improve hyperfibrinolysis and improve hyperfibrinolysis even when coagulation tests are not impaired and helps blood to coagulate in vitro.^20^ Intravenous erythropoietin was well tolerated in diffuse axonal injury (DAI)^10^ and acute ischemic stroke^20^ and was related to an improvement in patient's outcome. On the other hand, Nirulaet al. showed that erythropoietin did not decrease neuronal cell death in comparison with placebo. According to Nichol et al., erythropoietin did not change the total number of patients with proper recovery or moderate disability six months after moderate or severe TBI.^21^ A clinical trial on stroke showed no beneficiary influence of erythropoietin (greater mortality than the placebo-receiving control samples).^22^ Despite its anti-apoptotic effects and ability to reduce the inflammatory response, as well as its neurotrophic action, EPO is actively involved in neuroprotection in various neurological trauma types.^23^ A 2015 study by Devine on factors affecting the reduction of bleeding following thrombolytic therapy in patients with ischemic stroke concluded that cryoprecipitation, along with other factors including FFP and transhexamic acid, could partially prevent the spread. Bleeding caused by thrombolytic drugs.^24^ The important point in this study was that thrombolytic therapy with various drugs including (tPA) activated the anticoagulant system to remove thrombosis, which can cause coagulopathy to cause bleeding inside the brain tissue and ischemic stroke to hemorrhagic stroke. It has been shown that the administration of cryoprecipitate can prevent the spread of bleeding to some extent. Therefore, considering the similar mechanism of hemorrhage in the administration of thrombolytic and hemorrhage following trauma, it can be concluded that cryoprecipitate has the ability to prevent the spread of cerebral parenchymal tissue hemorrhage by modifying coagulopathy. The possible mechanism of cryoprecipitate action, which contains fibrinogen factors, VWF and factors 8 and 13, is that it acts both as a source of fibrinogen, which plays a direct role in fibrin formation for homeostasis, and contains factor 8, which also activates the coagulation pathway of the internal cascade and has the ability to prevent the growth of hematoma. The factor 13 through thrombin in the presence of calcium causes the stable binding of fibrin monomers and forms a stable clot. It should be noted that fibrinogen also plays an important role in platelet aggregation. One of the advantages of cryoprecipitate blood product is that it has a very low volume and does not require RH and ABO matching during injection and can be stored for a long time.

**Limitations:**

First, based on the results in the article, the authors speculated that early cryoprecipitate transfusion reduced the mortality rates of patients with isolated TBI by preventing coagulopathy development; however, it is not possible to definitively comment on the mechanisms behind our findings. Second, only acute transfusion-related adverse events were assessed in this study. Although a low rate of adverse events for cryoprecipitate has been reported, it should be noted that the incidence of long-term adverse events remains unclear, and transfusion has inherent risks such as unknown-virus infection. Third, the sample size of this study was small, and statistical power was low. Further research with larger sample size is therefore warranted. Finally, the patients in this study were older than those included in a previous study. Our results therefore warrant validation in other cohorts. Fourth, most of the cases in this study had mild TBI and based on our findings, we cannot extrapolate our findings to the severe cases of TBI. Another limitation of this study is the absence of people under 18 years of age and the small size of the study sample. Therefore, it is suggested to pay enough attention to these points in future studies.

## Conclusion

In this study, the effect of administering cryoprecipitate blood product on preventing the spread of cerebral parenchymal tissue bleeding in trauma patients was investigated. The findings of this study indicate that early administration of 4 units of cryoprecipitate blood product in patients with cerebral hemorrhage due to trauma can significantly prevent the spread of bleeding neural tissue and prevent further neurological damage.
